# miTAR: a hybrid deep learning-based approach for predicting miRNA targets

**DOI:** 10.1186/s12859-021-04026-6

**Published:** 2021-02-27

**Authors:** Tongjun Gu, Xiwu Zhao, William Bradley Barbazuk, Ji-Hyun Lee

**Affiliations:** 1 Bioinformatics, Interdisciplinary Center for Biotechnology Research, University of Florida, Gainesville, FL USA; 2 Division of Quantitative Sciences, University of Florida Health Cancer Center, University of Florida, Gainesville, FL USA; 3 Department of Ophthalmology and Visual Sciences, University of Michigan, Ann Arbor, MI USA; 4 Department of Biology, University of Florida, Gainesville, FL USA; 5 Genetics Institute, University of Florida, Gainesville, FL USA; 6 Department of Biostatistics, University of Florida, Gainesville, FL USA

**Keywords:** Deep learning, Hybrid model, MiRNA target, Convolutional neural networks, Recurrent neural networks

## Abstract

**Background:**

microRNAs (miRNAs) have been shown to play essential roles in a wide range of biological processes. Many computational methods have been developed to identify targets of miRNAs. However, the majority of these methods depend on pre-defined features that require considerable efforts and resources to compute and often prove suboptimal at predicting miRNA targets.

**Results:**

We developed a novel hybrid deep learning-based (DL-based) approach that is capable of predicting miRNA targets at a higher accuracy. This approach integrates convolutional neural networks (CNNs) that excel in learning spatial features and recurrent neural networks (RNNs) that discern sequential features. Therefore, our approach has the advantages of learning both the intrinsic spatial and sequential features of miRNA:target. The inputs for our approach are raw sequences of miRNAs and genes that can be obtained effortlessly. We applied our approach on two human datasets from recently miRNA target prediction studies and trained two models. We demonstrated that the two models consistently outperform the previous methods according to evaluation metrics on test datasets. Comparing our approach with currently available alternatives on independent datasets shows that our approach delivers substantial improvements in performance. We also show with multiple evidences that our approach is more robust than other methods on small datasets. Our study is the first study to perform comparisons across multiple existing DL-based approaches on miRNA target prediction. Furthermore, we examined the contribution of a Max pooling layer in between the CNN and RNN and demonstrated that it improves the performance of all our models. Finally, a unified model was developed that is robust on fitting different input datasets.

**Conclusions:**

We present a new DL-based approach for predicting miRNA targets and demonstrate that our approach outperforms the current alternatives. We supplied an easy-to-use tool, miTAR, at https://github.com/tjgu/miTAR. Furthermore, our analysis results support that Max Pooling generally benefits the hybrid models and potentially prevents overfitting for hybrid models.

## Background

microRNAs (miRNAs) are small regulatory RNAs that are ~ 22 nucleotides (nts) in length [1]. They typically form complementary hybrid sequences with their targets and act to repress gene expression or cleave mRNAs at the post-transcriptional level [1, 2]. It has been reported that miRNAs play key roles in a variety of biological processes and human diseases [3], including cell differentiation and development, metabolism, proliferation and apoptosis, viral infection, tumorigenesis, diabetes, macro- or micro-vascular complications, and neurological diseases. Thus, it is important to find the targets of miRNAs to better understand the function and regulation of miRNAs.

Advances in understanding of the interactions between miRNAs and their targets have led to the development of many computational methods/tools to predict miRNA targets. The majority of these tools are based on common features of the miRNA:target interaction. Four features are widely used: sequence complement (especially in the seed region that is generally defined as a 6 or 7 nts sequence starting at the second or third nucleotide (nt) of the miRNA sequence), thermodynamic stability, target site accessibility, and sequence conservation among species [4]. Several widely used tools have been developed based on these features. For example, miRanda [5] relies on sequence complementarity and binding energy; TargetScanS [6] relies on sequence complementarity in seed region; while PITA [7] relies on target site accessibility. However, miRNA targets predicted by different methods and tools are inconsistent with one another. Furthermore, using known features limits the ability to predict novel or non-canonical miRNA targets, which have been determined to be prevalent [8].

Recently, several deep learning (DL) methods were developed to handle unknown features and to improve the accuracy of prediction. MiRTDL [9] uses a convolutional neural network (CNN) to capture training sets features. Although CNNs can automatically assess feature importance, miRTDL is still based on known features. MiRTDL used 20 features from three categories: three conservative features, nine complementary features, and eight accessibility features. Consequently, information outside these several features cannot be captured. DeepTarget [10] uses deep recurrent neural network (RNN) based autoencoders to learn sequence features for miRNAs and genes separately, and then uses a stacked RNN to learn the sequence-to-sequence interactions between miRNA and their targets. It supplies a way to predict the target of miRNAs without using any pre-defined features. However, RNN may not be efficient in learning spatial features that exist in miRNA:target interactions. In 2018, two new DL methods were developed: DeepMirTar [11] and miRAW [12]. DeepMirTar collects a set of 750 features and uses stacked denoising auto-encoders for miRNA target prediction. Although DeepMirTar significantly increases the number of features, it still depends on features derived from the four major feature types mentioned previously. MiRAW includes three major steps: two filter steps were used before and after a deep feed forward neural network step. The input for miRAW are the sequences of the concatenated miRNA and its target, which avoids pre-definition of the features. However, a feed forward neural network may not be efficient to capture the spatial and sequential features of the hybrid sequences of miRNA:target [13].

Encouraged by the better accuracy and large training datasets collected from recent DL studies, we developed a novel DL-based approach that integrates two major types of neural networks, CNNs and RNNs, to predict miRNA targets. CNNs are designed to learn spatial features and RNN are designed to learn sequential features [13, 14]. By combining CNNs and RNNs, our approach has the advantages of learning both the intrinsic spatial and sequential features of miRNA:target. Recent studies in functional DNA sequence discovery have demonstrated that hybrid architectures outperform models utilizing solely CNNs or RNNs [15–17]. The inputs for our models are the primary sequences of miRNAs and genes. We trained two models on two datasets obtained from the studies of DeepMirTar and miRAW. We demonstrated that our models achieved higher accuracies relative to those reported in the DeepMirTar and miRAW studies. In addition, we obtained substantially better performance than the two studies on independent datasets. Thirty six of the 48 independent positive miRNA:target pairs were identified by our model, while DeepMirTar detected only 24 of the 48. Our model achieved an accuracy at 0.966 and specificity at 0.976, while miRAW achieved an accuracy at 0.913 and specificity at 0.363. We further compared our models with two earlier studies on smaller datasets, resulting in an overall better performance. In the end, a unified model was developed that can fit both DeepMirTar and miRAW datasets.

Besides model development, we examined the function of a Max pooling layer in hybrid models. Our results demonstrated that a Max pooling layer can improve the performance for all our models.

## Methods

### Datasets

We obtained two datasets that contain sequences of human miRNAs and genes from the studies of DeepMirTar and miRAW [11, 12]. The first dataset was downloaded from the Additional file tables of the DeepMirTar study, which contains 3915 positive pairs of miRNA:target and 3905 negative pairs of miRNA:target. The positive pairs in the DeepMirTar dataset were obtained from two resources: mirMark data [18] and CLASH data [19]. And only the target sites located in 3′UTRs, and the target sites with canonical seeds (exact W–C pairing of 2–7 or 3–8 nts of the miRNA) and non-canonical seeds (pairing at positions 2–7 or 3–8, allowing G–U pairs and up to one bulged or mis-matched nucleotide) were included. The negative pairs were generated by shuffling the real mature miRNAs. Details on generating the datasets are in the DeepMirTar study [11]. We first evaluated the dataset by examing whether the miRNA sequences are consistent with the latest miRBase (release 22) (http://mirbase.org/ftp.shtml). We removed the miRNAs that cannot be found from the current version of miRBase. Finally, a total of 3908 positive pairs and 3898 negative pairs were kept. The data were termed DeepMirTar (Table 1). In the DeepMirTar study, an independent dataset was collected from PAR-CLIP experiment (48 positive miRNA:target pairs), which was also used as an independent dataset in our study [20]. This set was termed DeepMirTarIn (Table 1).

**Table 1:** The number of miRNA:target pairs in each dataset used in training, validating and testing the proposed approach

Datasets	Positive pairs	Negative pairs	Training set	Validation set	Test set
DeepMirTar dataset	3908	3850	4964	1241	1552
miRAW dataset	31,660	30,993	40,096	10,025	12,531
MirTarRAW dataset^a^	13,860	13,860	17,740	4435	5544
DeepMirTarIn dataset^b^	48	NA			
miRAWIn dataset^b^	929	890			
DeepMirTarLeft dataset^c^	443	385			
miRAWLeft dataset^c^	21,265	20,598			

Blank box means not applicable. NA represents the data do not exist

^a^Dataset generated by combining the DeepMirTar and the miRAW dataset. Details are in “Methods” section

^b^Independent test dataset for evaluating different methods/models

^c^The surplus dataset from DeepMirTar and miRAW combining the two datasets. Details are in “Methods” section

In the miRAW study, Albert Pla et al. [12] collected a large amount of verified data that included both canonical and non-canonical miRNA:target pairs. The experimentally validated positive and negative miRNA:target pairs were collected from two resources: Diana TarBase [21] and MirTarBase [22], and the target site sequences were obtained by cross-referencing with PAR-CLIP [23], CLASH [19], and TargetScanHuman 7.1 [8]. In total, 32,726 positive and 31,992 negative pairs were collected. We also removed the miRNAs that are not consistent with the current version of miRBase. Finally, a total of 32,660 positive and 31,993 negative pairs were retained. In the miRAW study, a subset of the data was kept as an independent dataset that have no intersections with the training, test and validation datasets. Similarly, we randomly selected 2000 pairs and removed any data that were overlapped with the training, test and validation datasets, resulting 929 positive and 890 negative pairs as an independent test dataset. This set was labelled miRAWIn. The remaining pairs were labeled miRAW (Table 1).

In addition, we combined ~ 33% of miRAW data (20,790 pairs) and ~ 90% of DeepMirTar data (6930 pairs) into one dataset (termed MirTarRAW) for training a unified model. Because miRAW contains eight folds more data than the DeepMirTar, we included the majority of DeepMirTar (90%) in MirTarRAW while kept 10% for examining the performance of the unified model. To prevent the miRAW data dominating the combined dataset, we limited the number of miRAW to be not more than three folds of DeepMirTar, which resulted in ~ 33% of the miRAW to be included. The remaining data from DeepMirTar were taken as a test dataset and labelled DeepMirTarLeft; the remaining data from miRAW were also taken as a test dataset and labelled miRAWLeft (Table1).

For each dataset, we concatenated the sequences of miRNAs from 3′ to > 5′ with their target sequences from 5′ to > 3′. To keep all the miRNA sequences the same length, those miRNA sequences with length less than the longest miRNA (26 nts across both datasets) were padded with ‘N’s. The same padding was done for the target sequences, which had targets sites of up to 53 nts. All the target sites in the miRAW dataset were trimmed to the same length by Albert Pla et al*.* [12], which is 40 nts. After padding, the miRNA sequence and the target sequence were concatenated directly. Thus, the length of the sequences after concatenation for the DeepMirTar dataset is 79, and 66 for the miRAW dataset.

### Overview of the hybrid DL-based approach for predicting miRNA targets

**Fig. 1:**
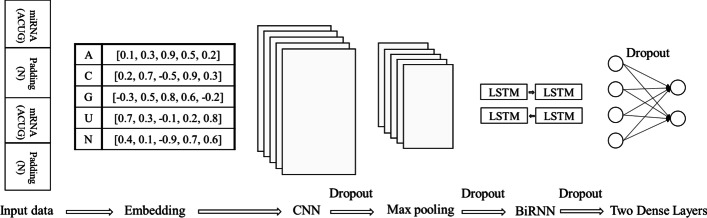
Overview of the proposed DL-based approach for predicting miRNA targets. As inputs of the approach, miRNA sequences and gene sequences are padded separately and then concatenated directly. The approach contains one embedding, CNN, Max pooling, and BiRNN layer, and two dense layers. To prevent overfitting, a dropout is added following CNN, Max pooling, BiRNN and between the two Dense layers

Six layers were used for miRNA target prediction (Fig. 1). Although our model is a hybrid model, it contains less layers than the models used in DeepMirTar (seven layers) and miRAW (eight layers). The first layer is an embedding layer. The embedding layer converts the input data into a five-dimensional dense vector that can be initialized randomly and trained with the other five layers. The second layer is a 1D-convolutional layer, which aims to learn the spatial features between miRNA:target. The third layer is a max pooling layer that normally follows the CNN layer to reduce the dimensionality of the input data. The fourth layer is a bi-directional RNN (BiRNN). The BiRNN can learn the sequential features of miRNA:target from the forward and reverse directions. The fifth and sixth layer are dense layers that were used to calculate the final classification. To reduce the probability of overfitting and to make the approach more generalized for predicting future cases, a dropout was added following the second layer (Fig. 1). The major elements of the approach are described below.

### The embedding layer

The one-hot encoding technique is widely used to transform the input sequences into numeric vectors. However, one-hot encoding normally generates sparse high-dimensional vectors that may affect the performance of the model [24]. The embedding layer can not only transform the sequences into dense vectors, but also can be updated along with all the other layers throughout the training process. It is reported that the embedding layer performs better than one-hot encoding [25]. Normally the size of the vector equals the vocabulary size. Since five different letters, {A, U, G, C, N}, were in our datasets, we transformed our input sequences into five-dimensional vectors with one vector for one letter in one input sequence. An example was shown in Fig. 1, for instance, for a sequence with length of ten, ten dense vectors are generated and the length for each vector is five.

### The CNN layer

CNN is a neural network that uses filters/kernels to scan the input data in order to capture the embedding spatial information [13, 14]. It has been widely used in image processing and recently also been applied in many biological and clinical data analyses. The parameters in a filter/kernel can be shared while scanning different regions of input data. Thus, the model parameters can be greatly reduced, which is one of the advantages to use CNN. In our model, a CNN layer was added following the first embedding layer. The number of kernels was set at 320 with the kernel size of 12. The nonlinear activation function, rectified linear unit (ReLU), was chosen in the CNN layer, which is more robust to gradient vanishing or gradient explosion. Specifically, the formula for calculating the output of the CNN layer is:

$${\text{Convolution}}\left( X \right)_{m}^{n} = w^{n} \cdot x = {\text{Re}} LU\left( {\mathop \sum \limits_{i = 0}^{I - 1} \mathop \sum \limits_{j = 0}^{J - 1} w_{i,j}^{n} *x_{m + i,j} } \right),$$

where w is the kernel, x is the encoded input sequence, m is the index of the output position, n is the index of the kernel, i = 0,…,I − 1, is the index of the kernel position, j = 0,…,J − 1, is the index of the input channel, I and J are the size of the kernel (12 in our study) and the number of the channel (five in our study) respectively. The · operator represents the element-wise product.

### The RNN layer

RNN is a type of neural networks used widely in natural language processing, speech and image recognition [13, 14]. In recent years, RNN has been applied in various biological fields. The design of RNN naturally fits sequential or time-series data and can model sequences of various length. The hidden layers of an RNN accept not only the input data from previous layers but also the output from the latest time point. A simple RNN can be expanded along the time series into a complicated network. Consequently, a simple RNN is prone to problems like gradient vanishing in the training process and it can be difficult to learn long term dependencies. A few advanced RNNs including long short-term memory (LSTM) and gated recurrent unit have been developed to solve these problems. Both approaches use memory based hidden units rather than simple perceptron hidden units, which greatly improve the performance. In our approach, we used the LSTM layer to learn the dependencies between miRNA:target. Since it is possible that the dependencies may exist in the order of target:miRNA, we used bidirectional LSTM (BiLSTM) to learn the sequential information from both directions. The size for the hidden units was set at 32. We also used the ReLU as the activation function for the BiLSTM. For each LSTM unit at time step t, the following operations were performed:

$$i_{t} = \sigma \left( {W_{i} x_{t} + V_{i} h_{t - 1} + b_{i} } \right),$$

$$c_{t}^{\sim } = \tanh \left( {W_{c} x_{t} + V_{c} h_{t - 1} + b_{c} } \right),$$

$$f_{t} = \sigma \left( {W_{f} x_{t} + V_{f} h_{t - 1} + b_{f} } \right),$$

$$c_{t} = f_{t} \cdot c_{t - 1} + i_{t} \cdot c_{t}^{\sim } ,$$

$$o_{t} = \sigma \left( {W_{o} x_{t} + V_{o} h_{t - 1} + b_{o} } \right),$$

$$h_{t} = o_{t} \cdot {\text{tanh}}\left( {c_{t} } \right),$$

where $i_{t}$, $f_{t}$, $o_{t}$ represent the three gates used in LSTM respectively: input gate, forget gate and output gate. W and V represent the weights for the input and the previous cell output. $b$ is the bias term. $\sigma$ represents the sigmoid function. $c_{t}^{\sim }$ represents the new values that can be added to the memory cell. $h_{t}$ represents the output. The $\cdot$ operator represents the element-wise product.

### Dropout

Overfitting is a major problem that DL methods face. Dropout is one method to prevent overfitting [14] and was used in our model. It discards some neuron units from the network according to a certain probability. A variety of probabilities were tested in model selection process. We applied a dropout following every layer, except the first embedding and the last layer.

### Evaluation metrics

We used the following metrics to evaluate the performance of our models: accuracy, sensitivity, specificity, F-measure (or F-score), positive predictive value (PPV), negative predictive value (NPV), and Brier Score. Accuracy examines how close the measured values to the true value. It is calculated as: accuracy = (TP + TN)/(TP + TN + FP + FN), where TP, TN, FP and FN represent the number of true positives, true negatives, false positives and false negatives, respectively. Sensitivity and specificity measure the power of the model for detecting true positives and true negatives, respectively. They were calculated as: sensitivity = TP/(TP + FN), specificity = TN/(TN + FP). PPV and NPV measure the precision of the model on correctly predicting the true positives and true negatives, respectively. They were calculated as: PPV = TP/(TP + FP), NPV = TN/(TN + FN). F-score is the harmonic mean of PPV (precision) and sensitivity (recall), which can be used as a parameter that integrates precision and recall and is more informative for measuring the performance of models. Brier Score measures the accuracy of probabilistic predictions. Rather than using the discrete prediction outcomes, Brier Score uses the prediction probability that is assigned to each outcome. It calculates the mean squared difference between the prediction probabilities and the true values. The 95% confidence intervals (CIs) for the metrics were calculated based on the 2.5% and 97.5% percentiles of the respective parameter.

### Code implementation and availability

Implementation of the approach was done in Python v3.6.5 using Keras with TensorFlow as the backend. Source codes are available at https://github.com/tjgu/miTAR. It takes about six hours to perform one-time training on the miRAW dataset on a desktop with four cores and 16 GB memory (Intel(R) Core(TM) i5-9500 CPU @3.00 GHz) and one and half hour on the DeepMirTar dataset. Thus, depending on the size of the dataset and the times of training, computational time varies significantly. We also supplied tools for predicting the targets of one or multiple miRNAs using our trained models, which only takes a few minutes to compute. The inputs for our tools are sequences of miRNAs and genes that are ordered from 5′ to > 3′. Our tool re-orders the miRNA sequence automatically from 5′ to > 3′ to 3′ to > 5′ to fit the model requirement. To assist the usage, we integrated codes to automatically split long gene sequences into overlapped short sequences that can fit the input of our different models. Furthermore, a few parameters can be adjusted based on the goal of the users’ work. For example, the probability for determining whether a short sequence is the binding site of a miRNA, and the number of target sites in a gene for determining whether the gene is a target gene. The outputs are saved as a fasta file containing the sequences of the targeted sites.

## Results

### Two models were trained on the DeepMirTar and miRAW datasets separately

We first trained our model on the two datasets separately, DeepMirTar and miRAW. To obtain the optimal model parameters, a wide range for each parameter was tested: the learning rates at 0.2, 0.1, 0.05, 0.01, 0.005, and 0.001; the dropout rates at 0.1, 0.2, 0.3, 0.4, and 0.5; and the batch sizes at 10, 30, 50, 100, and 200. The size of epoch was set at 1000. To prevent overfitting, we employed early stopping in addition to dropout. The program stops training when the accuracy of the model does not improve by 0.1% in 100 epochs.

We split DeepMirTar and miRAW datasets into three sets separately: 20% were used as a test dataset, 64% were used as a training dataset and 16% were used for a validation dataset (Table 1). For the DeepMirTar dataset, the parameters generated the highest accuracy are learning rate at 0.005, dropout at 0.2 and batch size at 30, which were chosen in the downstream analysis. The model trained with this set of parameters was labelled as miTAR1. Then we randomly split the DeepMirTar dataset 30 times into training, validation and test sets and ran the same model structure 30 times. We obtained an average accuracy of 97.9% (Table 2). The set of parameters that produced the highest accuracy for the miRAW dataset is learning rate at 0.1, dropout rate at 0.4 and batch size at 200. We randomly split the miRAW datasets 30 times similarly as the DeepMirTar dataset and obtained an average accuracy of 96.5% (Table 2). We labelled this model as miTAR2.

**Table 2:** Performance evaluation metrics for miTAR1 and miTAR2

Model: dataset	Accuracy	Sensitivity	Specificity	F-score	PPV^c^	NPV^c^	Brier score
DeepMirTar: DeepMirTarRaw^a^	0.9348	NA	NA	0.9348	NA	NA	NA
miTAR1: DeepMirTar Test set	0.9781	0.9648	0.9921	0.9783	0.9922	0.9641	0.0214
miTAR1: DeepMirTar (30 times)^b^ [95% CI]	0.9787 [0.9714–0.9836]	0.9717 [0.9615–0.9801]	0.9857 [0.9759–0.9921]	0.9786 [0.9717–0.9837]	0.9858 [0.9756–0.9922]	0.9719 [0.9610–0.9807]	0.0193 [0.0144–0.0265]
miRAW: miRAWRaw^a^	0.935	0.935	0.938	0.935	NA	NA	NA
miTAR2: miRAW Test set	0.9654	0.9609	0.9697	0.9652	0.9695	0.9613	0.0283
miTAR2: miRAW (30 times)^b^ [95% CI]	0.9649 [0.9601–0.9686]	0.9616 [0.9562–0.9678]	0.9683 [0.9618–0.9740]	0.9651 [0.9604–0.9693]	0.9687 [0.9623–0.9742]	0.9610 [0.9558–0.9676]	0.0271 [0.0246–0.0296]

NA represents the value is not reported in the corresponding study

^a^DeepMirTarRaw and miRAWRaw present the dataset used in the DeepMirTar and the miRAW study. The best performance values are selected for the performance metrics if multiple values are reported in the respective study for different conditions

^b^Evaluation was done by randomly running on the DeepMirTar and the miRAW datasets 30 times. The average value and the 95% confidence interval (given in []) were reported here. Details are in “Results” section

^c^PPV represents positive predictive value; NPV presents negative predictive value

### Performance comparison with the two studies of DeepMirTar and miRAW using test datasets

We compared the performance of miTAR1 and miTAR2 with the two studies, DeepMirTar [11] and miRAW [12]. Our datasets, DeepMirTar and miRAW, were obtained from the same studies and they were split at a similar proportion: 60% and 66.7% of the data were used as the training datasets in the DeepMirTar and miRAW studies respectively, while 64% were used in our study. Therefore, it is worthy to note that we did not re-run DeepMirTar and miRAW for the comparison. We selected the best overall performance (also the best accuracy) reported in the two studies for comparison with miTAR1 and miTAR2. Our models achieved higher accuracies than the DeepMirTar (97.9% vs 93.5%) and miRAW (96.5% vs 93.5%) studies. The results were shown in Table 2.

In addition to the accuracy, additional metrics also showed better performance of our models than the models reported in the DeepMirTar and miRAW studies, and detailed results of sensitivity, specificity, F-measure (or F-score), PPV, NPV, and Brier Score are present in Table 2.

### Performance comparison with the two studies of DeepMirTar and miRAW using independent datasets

Furthermore, we compared our results using the independent test datasets with the studies of DeepMirTar and miRAW. The positive pairs (48 pairs) in the independent dataset (DeepMirTarIn) are the same as the dataset used in the DeepMirTar study, which was obtained from an independent source. We identified 36 of 48 positive miRNA:target pairs, which is 50% more than the 24 reported in the DeepMirTar study (Table 3). To compare with the miRAW study, 929 positive and 890 negative pairs from the miRAW dataset were excluded from the training dataset and taken as an independent dataset (miRAWIn). They were obtained in the same way as the miRAW study but balanced between the positive pairs and negative pairs. We obtained an accuracy of 96.9 on the dataset of miRAWIn, which is higher than the accuracy of 91.3 reported in miRAW (Table 4). The miRAW study also reported the sensitivity (93.1%), specificity (36.3%) and F-score (95.4%) for the test on the independent dataset, which are all lower than our model (sensitivity: 96.0%; specificity: 97.9%; and F-score: 96.9%), especially for the specificity (Table 4).

**Table 3:** Performance comparison between miTAR1 and DeepMirTar on an independent dataset

Model: dataset	Identified positive miRNA:targets/Total number of positive pairs (%)
DeepMirTar: DeepMirTar Independent test set	24/48 (50%)
miTAR1: DeepMirTarIn	36/48 (75%)

The positive pairs for both models are the same set of pairs

**Table 4:** Performance comparison between miTAR2 and miRAW on an independent test dataset

Model:dataset	Accuracy	Sensitivity	Specificity	F-score	PPV^b^	NPV^b^	Brier score
miRAW: miRAWRaw^a^	0.913	0.931	0.363	0.954	NA	NA	NA
miTAR2: miRAWIn	0.966	0.957	0.976	0.967	0.977	0.956	0.028

NA represents the value is not reported in the corresponding study

^a^miRAWRaw presents the dataset used in the miRAW study. The best performance values were selected from the reports of the miRAW study under different conditions

^b^PPV represents positive predictive value; NPV represents negative predictive value

### Performance comparison with earlier studies using small datasets

Beside DeepMirTar and miRAW, we compared our approach to two other published DL methods: deepTarget and miRTDL [9, 10]. They were developed earlier, hence, the training datasets they used were smaller. 507 target site-level and 2891 gene-level miRNA:target pairs from mirMark repository [18] were taken as the positive training dataset in the deepTarget study. The negative pairs (507 site-level and 3133 gene-level) were generated by shuffling the real miRNA seed sequences [9]. mirMark repository was part of the DeepMirTar dataset, subsequently, we randomly extracted the same account of data from DeepMirTar dataset to train a model for comparing with deepTarget. 2891 positive and 3133 negative pairs from DeepMirTar dataset were used. The process for training the model is the same as miTAR1 and miTAR2. The trained model is labelled miTAR3. Although deepTarget performs better on NPV (97.6% vs 98.5%), our model outperforms the deepTarget model on accuracy, sensitivity, specificity, F-score, and PPV, especially the PPV (98.4% vs 88.5%) and F-score (97.9% vs 91.1%) (Additional file 1: Table S1), indicating miTAR1 is much more effective on miRNA target prediction than deepTarget.

miRTDL extracted 1297 positive and 309 negative miRNA and gene pairs from TarBase [21] database as the starting point to obtain 19,000 positive and negative site-level miRNA:target pairs for training their model [10]. The TarBase is one of the sources for generating the miRAW dataset. Accordingly, we trained a model using the same amount of data from miRAW dataset to compare with miRTDL. The process is the same as training miTAR1, miTAR2 and miTAR3, and the new model is labelled miTAR4. miRTDL only reported their results for accuracy, sensitivity and specificity. Although miRTDL outperforms on sensitivity (95.5% vs 96.4%) slightly, our model performs better on accuracy (96.0% vs 90.0%) and specificity (96.5% vs 88.4%) (Additional file 1: Table S2). In summary, the results support that our approach is more robust on small dataset than deepTarget and miRTDL.

### Performance evaluation of the unified model, miTAR

Since the length of the input sequences from DeepMirTar (79 nts) and miRAW (66 nts) datasets is different, miTAR1 cannot be fit by miRAW dataset, and miTAR2 cannot be fit by DeepMirTar dataset. To build a unified model that is more robust to handle different length of the input sequences and to save the users’ effort on choosing a model, we combined the two datasets and trained a new model that can fit both datasets. Due to much more pairs of miRNA:target in the miRAW dataset, we only randomly extracted ~ 33% of miRAW data, which contains 10,395 positive and negative data respectively. The majority of the DeepMirTar data were extracted (90%), which contained 3,465 positive and negative data separately (Details are in “Methods” section). We labelled the combined dataset as MirTarRAW (Table 1). The MirTarRAW dataset were then split into three sets: 20% for testing, 64% for training, and 16% for validation. The remaining data from the DeepMirTar were labelled as DeepMirTarLeft; and the remaining data from the miRAW were labelled as miRAWLeft, which were used as additional test datasets. The best model parameters for the combined dataset, MirTarRAW, are learning rate at 0.005, dropout rate at 0.2, and batch size at 100. We randomly split the dataset 30 times and obtained an average accuracy of 95.5%, which is higher than the accuracies reported in the DeepMirTar (93.5%) study and the miRAW (93.5%) study. We further tested the model using the two additional datasets: DeepMirTarLeft and miRAWLeft. The accuracies obtained from both datasets are higher than the reports from either DeepMirTar or miRAW (Table 5). Lastly, we examined the performance of miTAR using the two independent datasets, DeepMirTarIn and miRAWIn. Substantial better performances were obtained: 93.8% (miTAR) vs 50.0% (DeepMirTar); 95.1% (miTAR) vs 91.3% (miRAW) (Table 5). We also observed consistent better results for all the other metrics, including sensitivity, specificity, and F-Score (Table 5). We labelled this model as miTAR.

**Table 5:** Performance evaluation metrics for the unified model, miTAR, using MirTarRAW test datasets and two independent test datasets

Model: dataset	Accuracy	Sensitivity	Specificity	F-score	PPV	NPV	Brier score
miTAR: MirTarRAW Test set	0.9627	0.9591	0.9663	0.9627	0.9664	0.9589	0.0321
miTAR: MirTarRAW (30 times)^a^ [95% CI]	0.9549 [0.9496–0.9610]	0.9538 [0.9418–0.9629]	0.9560 [0.9428–0.9657]	0.9548 [0.9489–0.9610]	0.9559 [0.9443–0.9662]	0.9540 [0.9424–0.9623]	0.0393 [0.0340–0.0438]
miTAR: DeepMirTarLeft	0.9770	0.9706	0.9844	0.9783	0.9862	0.9668	0.0200
miTAR: miRAWLeft	0.9476	0.9500	0.9452	0.9485	0.9471	0.9482	0.0440
miTAR: DeepMirTarIn	0.9375	0.9375	NA^b^	NA^b^	NA^b^	NA^b^	0.9254
miTAR: miRAWIn	0.9505	0.9494	0.9517	0.9514	0.9535	0.9474	0.0416

^a^Evaluation was done by randomly running on the MirTarRAW datasets 30 times. The average value and the 95% confidence interval (given in []) are reported here. Details are in “Results” section

^b^Because DeepMirTarIn only contains positive miRNA:target pairs, the specificity, F-Score, PPV and NPV cannot be calculated. NA represents the value is not available

### Max pooling layer improves the performances of all our trained models

It is still not clear whether a pooling layer is beneficial in hybrid models in biological studies. Some of the previous hybrid models connected CNNs directly with RNNs [15, 17]. In our model, we included a Max pooling layer in between a CNN and an RNN with the reasoning to potentially reduce overfitting. To test whether the Max pooling layer benefits our models, we removed the Max pooling layer from the three models and tested 30 times to obtain a confident performance evaluation (miTAR1, miTAR2 and miTAR). We found that the average accuracy for all the models were reduced and the reduction was larger for a smaller dataset (Additional file 1: Table S3). DeepMirTar dataset was used to train miTAR1 model, which was the dataset with the least number of miRNA:target pairs (7758). After removing the Max pooling layer, the accuracy for miTAR1 reduced from 97.9 to 88.8% (~ 9.1% reduction). miTAR was trained using MirTarRAW dataset, which contained 27,720 miRNA:target pairs. The accuracy for miTAR was reduced from 95.5 to 91.4% (~ 4.1% reduction) after excluding the Max pooling layer. miTAR2 was trained by the largest dataset, miRAW (62,653), and received the least reduction in accuracy (~ 1.2%) from 96.5 to 95.3%. Our results indicate that a Max pooling layer can generally increase the accuracy of DL models, especially for models trained on smaller datasets.

In addition, we performed the same set of parameter selection for the models without a max pooling layer: the learning rates at 0.2, 0.1, 0.05, 0.01, 0.005, and 0.001; the dropout rates at 0.1, 0.2, 0.3, 0.4, and 0.5; and the batch sizes at 10, 30, 50, 100, and 200. The parameter selection process is the same as we did for training the models of miTAR1, miTAR2 and miTAR. The model trained on the DeepMirTar was labelled miTAR1_noMP, the model trained on the miRAW dataset was labelled miTAR2_noMP and the model trained on MirTarRAW was labelled miTAR_noMP. The parameters generated the highest accuracy for the miTAR1_noMP model are learning rate at 0.1, dropout rate at 0.3 and batch size at 100; for the miTAR2_noMP model are learning rate at 0.01, dropout rate 0.3 and batch size at 200; and for the miTAR_noMP are learning rate at 0.005, dropout rate at 0.2 and batch size at 30. We also performed 30 times’ training as we did for miTAR, miTAR1 and miTAR2. The average accuracies for miTAR_noMP (91.2%), miTAR1_noMP (97.5%), and miTAR2_noMP (94.3%) are all lower than the accuracies for miTAR, miTAR1, and miTAR2 (Additional file 1: Table S4). Furthermore, we added the max pooling layer back to the miTAR_noMP, miTAR1_noMP, and miTAR2_noMP at the same parameter setting and evaluated the performance by running 30 times’ training. We obtained higher accuracies for all the three models with a max pooling layer than without the max pooling layer (miTAR1_noMP: 97.9% vs 97.5%; miTAR2_noMP: 96.5% vs 94.3%; miTAR_noMP: 96.5% vs 91.2%) (Additional file 1: Table S4). The higher accuracies for all the models indicate that the max pooling in hybrid models generally benefits the performance.

## Discussion

We developed a hybrid DL-based approach that integrated two major types of neural networks, CNN and RNN, to predict the targets of miRNAs at a substantially higher accuracy than previous methods/tools on independent datasets. Using multiple model evaluation metrics, we demonstrated that our hybrid method significantly outperforms the latest DL methods, DeepMirTar and miRAW. Another advantage of our method is that the models do not require pre-defined features, which can save users’ efforts from calculating a large number of features. We also supplied a unified model that is more flexible on input datasets. Furthermore, we examined the contribution of a Max pooling layer in hybrid models and demonstrated that a Max pooling can significantly improve the performance for all of our models.

Our study is also the first study to perform comparisons across multiple DL-based approaches. We used the highest accuracy or the best overall performance reported from the respective DL studies to compare with the average performance of our models. We attempted to run the software from respective studies using the datasets we obtained, however, neither the software is available, nor the methods fit our datasets. We cannot obtain the miRTDL software from the website listed in the miRTDL study (http://nclab.hit.edu.cn/ccrm) [9]. deepTarget did not supply the source code for training the model (http://data.snu.ac.kr/pub/deepTarget) [10]. Nevertheless, the datasets used for miRTDL and deepTarget are theoretically subsets of miRAW and DeepMirTar. Thus, we trained two models with the same amount of data from miRAW and DeepMirTar. We demonstrate that our approach supplies an overall better performance than miRTDL and deepTarget, indicating our approach is more robust on small datasets. DeepMirTar used 750 features to train their model, but the 750 features were not supplied (https://github.com/Bjoux2/DeepMirTar_SdA) [11]. Regardless, we used a similar amount of data as the DeepMirTar study for training: 64% were used for training our model while 60% were used in the DeepMirTar study. In addition, we used the same independent dataset as the DeepMirTar study for comparison and we identified 50% more correct pairs (Table 3). We used miRAW dataset in a similar way as the original study: 64% were used for training in our study, while 66.67% were used in the miRAW study; we did 30 times’ training as the miRAW study did [12]. We selected the best value of all miRAW’ tests to compare with the average value of our model. Therefore, our results are comparable to the results reported in studies of DeepMirTar and miRAW. We demonstrate that our approach outperforms DeepMirTar and miRAW substantially on independent datasets.

The four DL studies (DeepMirTar, miRAW, deepTarget and miRTDL) we illustrated earlier did not perform comparisons across the DL studies, but conducted comparisons with non-DL methods [9–12]. They demonstrate the DL-based approaches outperform the non-DL methods. deepTarget compared with MBSTAR, miRanda, PITA, RNA22, TargetScan and TargetSpy, and proves that deepTarget delivers an unprecedented increase on accuracy [10]. Similarly, miRTDL shows that the non-DL methods (MiRTif and NBmiRTar) are far less accurate than miRTDL [9]. In addition to test dataset, DeepMirTar collected an independent dataset and presents that DeepMirTar is the best approach on identifying the correct pairs of miRNA:target than miRanda, RNAhybrid, PITA, TargetScan v7.0, and TarPmiR [11]. Similarly, miRAW demonstrates that it generally significantly outperforms the non-DL methods (TargetScan v7.1, PITA, mirSVR, mirDB, microT, Paccmit, and mirza-G) on an independent dataset using multiple evaluation metrics except specificity [12]. In summary, the four studies deliver that the DL-based approaches supply substantial improvements on performance than the non-DL methods. Although we did not compare directly with non-DL methods, our approach outperforms the four DL-based approaches, indicating our approach has a high probability to outperform the non-DL methods.

When miRNAs bind to the genes, they form hybrid secondary structures. Spatial features exist in miRNA:target. The CNN is designed to capture the spatial features. Therefore, theoretically, models with a CNN perform better. We did a simple test to examine this. We removed the steps that associated with CNN, for example, the CNN and max pooling layers. After removing the two layers and the associated dropouts, we observed poorer performance for all our models. For example, the accuracy dropped from 95.5 to 85.5% for the miTAR model (Additional file 1: Table S3). Although performance may increase significantly by changing other parameters, for example, the batch size and the number of units in the remaining layers, our results support the importance of spatial features in miRNA target prediction. One previous study, the miRTDL study, applied CNN in miRNA target prediction, however, the authors only used CNN as a classifier [9]. The miTAR model is the first to use CNN to capture the potential spatial features directly from the sequences of miRNAs and genes.

In addition to CNN, we tested whether other layers contribute positively to the performance of our model, including the fourth layer (RNN), the fifth layer (dense layer), and all the dropouts. We removed one type of the layers each time while keep all the other parts the same. The results were shown in Additional file 1: Table S5. The accuracies for the miTAR, miTAR1 and miTAR2 models from the three tests were reduced: the accuracy for the dropouts reduced most; the dense layer reduced least; and the RNN layer lies in between. Our results support that all the layers positively contribute to the performance of our models. Comparing to the same tests on the CNN layer and the max pooling layer (Additional file 1: Table S3), the CNN and the max pooling layer affect the accuracy of the models most, indicating spatial features play important roles in miRNA:target interactions.

The accuracy for all our models was higher than 95.5%, which indicates that our models can learn the intrinsic features between miRNA:target. However, understanding the features from the millions of parameters of the model is complicated, especially for our models with input of sequences. Unlike the models using pre-defined features, we cannot alter one of the pre-defined features to test the importance of each feature. Even so, many known features were reported that are important for a miRNA recognizing its target genes. We can correlate the known features with the output of each layer and validate the importance of the known features. In addition, by scrutinizing the sequences of miRNAs and genes from the positive and negative datasets, new features may be revealed. Attempts to decode the information learned from DL models are emerging and will be considered in our future work.

Although the performance of our models is higher than existing DL-based methods, one way we can improve is to quantitatively measure the effects of miRNAs on their targets. We plan to integrate the features learned from our models and gene expressions from a specific tissue/disease to supply quantitative measurements on miRNA’s targets. For example, rank the targets of miRNAs from the most function or diseases related to the least related. We believe the quantitative measurements can better assist the understanding of miRNA’ s function and further benefit the development of treatment on diseases.

## Conclusions

miRNAs modulate a broad range of essential cellular processes linked to human health and diseases, thus, identifying miRNA targets is critical for understanding miRNA’s function and treating miRNA associated diseases. Here we developed a new DL-based approach for miRNA target prediction. With multiple evidences and evaluation metrics, we prove that our approach outperforms other state-of-the-art DL methods. We supplied an easy-to-use tool for predicting miRNA target at https://github.com/tjgu/miTAR, which will benefit the research on studying miRNA function.

## Supplementary Information

**Additional file 1: Table S1.** Performance comparison with a previous developed DL method, deepTarget. **Table S2.** Performance comparison with a previous developed DL method, miRTDL. **Table S3.** Performance examination before and after removing the Max pooling layer or CNN and Max pooling layers. **Table S4.** Performance examination for models without the Max pooling layer at the optimal parameter settings. **Table S5.** Performance examination before and after removing one type of layer.

## Data Availability

The raw data used in our study are available from the two studies [11, 12]. The source codes and the tool are available at https://github.com/tjgu/miTAR.

## Abbreviations

CNNs: Convolutional neural networks
RNNs: Recurrent neural networks
DL: Deep learning
BiRNN: Bi-directional RNN
LSTM: Long short-term memory
PPV: Positive predictive value
NPV: Negative predictive value
TP: True positives
TN: True negatives
FP: False positives
FN: False negatives
CIs: Confidence intervals
